# Is a Handful an Effective Way to Guide Nut Recommendations?

**DOI:** 10.3390/ijerph18157812

**Published:** 2021-07-23

**Authors:** Rachel Brown, Andrew R. Gray, Mei Gee Chua, Lara Ware, Alex Chisholm, Siew Ling Tey

**Affiliations:** 1 Department of Human Nutrition, University of Otago, P.O. Box 56, Dunedin 9054, New Zealand; chume385@student.otago.ac.nz (M.G.C.); lara.ware@otago.ac.nz (L.W.); alex@dietdesign.co.nz (A.C.); siewling.tey@otago.ac.nz (S.L.T.); 2 Biostatistics Centre, Division of Health Sciences, University of Otago, P.O. Box 56, Dunedin 9054, New Zealand; andrew.gray@otago.ac.nz

**Keywords:** nuts, portions, serving sizes, handful, guidelines, recommendations

## Abstract

Dietary guidelines recommend consuming 30 g of nuts per day to reduce the risk of chronic disease. A ‘handful’ is commonly used to guide consumers. Research is lacking on how this translates into actual gram amounts. This study quantified the grams of nuts represented by different portion size measures, including a ‘handful’ and ‘30 g serving’ among 120 participants. Each participant was randomised to a sequence where they received three of six different nut types (from almonds, cashews, hazelnuts, macadamias, peanuts, and walnuts) and were instructed to take a: ‘usual serving’, ‘handful’, ‘small handful’, ‘large handful’, and ‘30 g serving’ of each. Combining all nut types, the median ‘handful’ was 36.3 g, compared to 28.7 g for the estimated ‘30 g serving’ and 24.8 for the ‘usual serving’. The ‘large handful’ was approximately double the ‘handful’ (61.3 g), whereas the ‘small handful’ was about half (16.7 g). Eighty-three percent of portions chosen were at least 80% of the recommended 30 g intake when participants were asked to take a ‘handful’, compared to 63% for the ‘30 g serving’. It appears a ‘handful’ can be used as a practical tool to guide recommended nut intakes, and increases the amount selected compared to instructions to take a ‘30 g serving’.

## 1. Introduction

Regular nut consumption is associated with a reduction in chronic disease, in particular cardiovascular disease [1,2,3,4]. A meta-analysis reported the relative risk reduction per 28 g/day increase in nut intake was 21% for cardiovascular disease and 22% for all-cause mortality [1]. To obtain health benefits, nuts must be consumed regularly and in sufficient amounts. To this end, many guidelines recommend the daily consumption of 30 g (or 1 ounce = 28.4 g) of nuts as part of a cardioprotective diet [5,6,7]. For example, the eating and activity guidelines for New Zealand (NZ) adults recommend eating a variety of nuts, and replacing less healthy snack foods with 30 g of nuts per day [6]. In order to provide a practical guide for quantifying the recommended 30 g portion, it is widely proposed that this is equivalent to one handful of nuts [8]. Although this is a simple and practical guide, it is unclear what this equates to in reality, and whether it is a useful guide to help individuals achieve the recommendation of 30 g of nuts per day.

Portion size estimates are also important when interpreting data from semi-quantitative food frequency questionnaires (FFQs), which are used to assess the dietary intakes of large groups [9]. These dietary assessment tools use standardised serving sizes, which can specify an amount such as 1 ounce [10], or use more subjective portions such as a ‘medium’ serve [11]. For this form of dietary assessment to be meaningful, participants need to have some understanding of what these serving sizes constitute. If they are interpreted incorrectly, nut consumption and nutrient intakes could be under- or over-estimated.

Although hands in various forms (e.g., fist, palm, finger width, handful) have been recommended as tools for the lay public to estimate food portions [8], there are surprisingly few studies which have examined this objectively [12]. Gibson et al. found that despite a fist equating to around 250 mL (i.e., a cup), portions estimated using a fist significantly underestimated the actual portion of a food [12]. However, they found that finger widths proved useful for certain geometrically (triangular prism) shaped foods such as cheese and pizza. A study in young adults reported that when participants were asked to serve a typical portion of food, and then estimate this portion, mean estimates for most foods were higher than actual amounts [13,14]. Overall, there is evidence that people find it inherently difficult to estimate portion size [12,13,14].

There is a distinct lack of research on the use of handfuls to help guide the public to meet nutrition recommendations, and overall, people struggle to quantify portion size. Therefore, we aimed to quantify the gram amounts of nuts represented by a ‘handful’ (including ‘large’ and ‘small’ variants), or when asked to select a ‘30 g serving’, or a ‘usual’ serving in the general adult population. Gaining an understanding of the gram amount calculated using these measures, as well as the magnitude of inter-individual variation, will allow us to determine whether these are useful portion guides for nut consumption.

## 2. Materials and Methods

### 2.1. Design

This was an experimental study to determine the weight of portions that individuals chose when they were asked to take five different portions described below. We included six different nut types, specifically almonds, cashews, hazelnuts, macadamia nuts, peanuts, and walnuts. These are commonly consumed nuts in NZ and internationally, and represent a variety of nut forms and shapes (e.g., pecans are, to some extent, morphologically similar to walnuts). All nuts were raw, whole (except for walnuts, which were presented as halves, given that this is how they are usually packaged), and were unsalted and unflavoured.

### 2.2. Participants

We recruited participants aged 18 years and older from the general public in Dunedin, NZ. The recruitment process involved posting advertisements on the internet (including social media), and on bulletin boards around the University and supermarkets. We aimed to recruit equal numbers of males and females (approximately 60 each), with a range of normal, overweight, and obese individuals (approximately forty in each category). We also aimed to recruit around forty individuals aged less than 30 years, forty aged between 30 and 59 years, and forty aged ≥ 60 years. Exclusion criteria included those who were allergic or intolerant to nuts.

All participants provided informed consent and ethical approval was obtained from the University of Otago Ethics Committee (D17/416).

### 2.3. Protocol

Participants attended one visit at the Human Nutrition clinic at the University of Otago. They firstly completed a short questionnaire including demographics (age, gender, and ethnicity) and questions designed to capture information on usual nut consumption (allowing us to calculate usual grams consumed per week for each nut type). We also asked a question about current hunger, as this may influence serving size. For this, we asked participants to rate their overall hunger on a 100 mm scale anchored with ‘Not at all hungry’ on the left side (0 mm) and ‘Extremely hungry’ on the right side of the scale (100 mm).

Participants were then asked to take from a large bowl of nuts: (1) what they perceived as a ‘usual serving’ of nuts, (2) a ‘handful’ of nuts, (3) a ‘small handful’ of nuts, (4) a ‘large handful’ of nuts, and (5) what they believed is equal to 30 g of nuts (an estimated ‘30 g serving’—a commonly used descriptor of a ‘serving’). After each participant completed the experiment, each of the portions was weighed on electronic dietary scales (Salter Electronic, Salter Housewares, Ltd., Kent, UK) by the investigators. In order to reduce participant burden and boredom, each participant was asked to take the five different portions for only three of the six different nut types. Each participant was randomised to a sequence of three different nut types according to a balanced randomisation scheme stratified by gender, age (3 levels: <30 years; between 30 and 59 years; and ≥60 years) and estimated BMI (based on self-reported height and weight, 3 levels: normal; overweight; and obese). The randomisation was also balanced for order for choosing the ‘large’ and ‘small’ handfuls.

Following the portion size exercise, height was measured using a stadiometer (Holtain Ltd., Crymych, UK) and weight was measured on electronic scales to the nearest 0.1 kg (Seca, Hamburg, Germany). We did not measure hand size because we wanted to minimise participant burden. Instead, we used measured height, as this has been shown to correlate with hand size [15,16].

### 2.4. Calculation of Contribution of Nuts from Different Portion Estimates to Total Energy Expenditure (TER)

The energy requirement for each participant was calculated using the FAO/WHO/UNU equation [17], and the physical activity level (PAL) for light activity. The energy content from each portion estimate for each nut type was calculated and expressed as a percentage of TER.

### 2.5. Sample Size

As between- and within-participant variability for measurement tasks was unknown, sample size calculations are presented in terms of standard deviations (SDs) to give an illustration of the study’s power for nut type–amount combinations. Having *n* = 60 participants for each nut type would allow us to estimate 95% confidence intervals for each nut-based task: ± 0.26 SDs overall (with the half-width representing a “small” effect size), ±0.38 for each gender (with the half-width representing a “small-medium” effect size), and ±0.47 (with the half-width representing a “moderate” effect size) for each age or BMI group. This will also provide 80% power to detect differences of 0.74 SDs (a “large” effect size) between the two genders and 0.91 SDs (a “huge” effect size) between any two of the three age or BMI groups using two-sided tests at the 0.05 level. While smaller differences could be of interest at the individual level, as much nut research is performed at the population level, small differences in amounts are unlikely to influence results in that context.

### 2.6. Statistical Analysis

Statistical analyses were mainly descriptive. Formal analyses of the quantities participants selected were performed using linear mixed models for each measurement task (the five serving sizes) with fixed effects for nut type (of the six possibilities), the sequence of the measurement task (first, second, or third nut type to accommodate learning and/or fatigue), gender, age, BMI, height, hunger rating, and frequency of nut intake (how often they consumed that nut type), along with a random effect for participant to accommodate clustering at this level. Standard model diagnostics were investigated. Analyses were performed using Stata 16.1 with two-sided 95% confidence intervals presented and two-sided tests performed at the 0.05 level.

## 3. Results

### 3.1. Participants

We recruited 124 participants from the general population in Dunedin, NZ. We were able to use data from 120 participants. Data from four participants were removed due to errors in the sequences as administered not matching their randomised sequence. The characteristics of participants are presented in Table 1. There was a higher proportion of females, and the (geometric) mean BMI was at the upper end of the healthy range. We had a high proportion of NZ Europeans and Asian participants.

### 3.2. Nut Quantities Selected for the Different Portion Measures

There was considerable variability in the range of estimates. For example, across all nut types, the range for a ‘handful’ was 9.1 g to 106.3 g. For the estimated ‘30 g serving’, the range was 6.0 g to 148.5 g.

Table 2 shows the median (interquartile range (IQR)) gram amounts of nuts selected by participants when asked to take the different portion measures. Among all participants and nut types combined, the median for the ‘handful’ was 36.3 g, compared to the estimated ‘30 g serving’ where the median was 28.7 g. The large handful’ (61.3 g) was approximately double the ‘handful’, whereas the ‘small handful’ (16.7 g) was about half. The ‘usual serving’ was 24.8 g.

There were some differences between nut types. Median ‘handfuls’ ranged from 30.9 g for walnuts to 41.1 g for cashews, all being above the recommended 30 g serving size. From the regression analyses, pairwise comparisons indicated the amounts for almonds, cashews, and macadamias were higher than hazelnuts, peanuts and walnuts.

The median amount for a ‘large handful’ ranged from 52.9 g for hazelnuts to 70.8 g for cashews, whereas a ‘small handful’ ranged from 14.6 g for peanuts to 19.6 g for macadamia nuts. For all nut types, the median ‘large handful’ was around double the recommended 30 g serving size, whereas the median for the ‘small handful’ was around half. From the regression analyses, we noted some differences between nut types, as shown in Table 2.

When participants were asked to select an estimated ‘30 g serving’, the median amounts ranged from 25.6 g for hazelnuts to 31.1 g for macadamias. The median serving was below 30 g for all nut types other than macadamias, although for cashews, this was only by 0.5 g. From the regression analyses, pairwise comparisons indicated that amounts for hazelnuts were lower than all others except walnuts, and that hazelnuts and walnuts were lower than macadamias and peanuts.

The median gram amount when participants were asked to take a ‘usual serving’ ranged from 16.9 g for hazelnuts to 33.0 g for cashew nuts. From the regression analyses, we noted some differences between nut types, as shown in Table 2.

There were some gender differences. Numerically speaking, all ‘usual serving’ gram amounts were higher in males compared to females, with the greatest difference for peanuts, which was 45% higher in males. All ‘handful’ amounts were 5.9% to 21.7% greater among males compared to females, with the exception of almonds, which were 7.2% higher in females. The ‘large handful’ was 7.2% to 27.9% higher for males than females over all nut types. The ‘small handful’ was 4.5% to 16.9% higher among males for all nut types, except for cashews, which were 1.6% higher in females. The estimated ‘30 g serving’ gram amounts for females were higher for almonds (12.5%) and peanuts (9.3%), with less than a 2% difference for all other nut types.

### 3.3. The Percent of Participants Choosing at Least 80% of the Recommended Intake with the Different Portion Measures

Among all participants when combining all nut types, 83.0% of participants chose a portion that was at least 80% of the 30 g recommended intake when asked to take a ‘handful’, compared to 62.7% and 52.0% when asked to take a ‘30 g serving’ and a ‘usual serving’, respectively (Table 3). When asked to take a ‘large handful’ and a ‘small handful’, 98.1% and 24.0% of participants, respectively, took portions of at least 80% of the recommendation.

There were some numerical differences by nut type. For example, when asked to take a ‘handful’, 90% of participants chose an amount equal to at least 80% of the recommendation for almonds and macadamias, whereas only 78.3% and 72.9% did so for hazelnuts and walnuts, respectively. For the estimated ‘30 g serving’, 53.3% chose amounts equal to at least 80% of the recommendation for almonds, compared to 71.7% of participants for cashews.

There were some gender differences. When nut types were combined, for ‘usual serving’, ‘handful’, and ‘large handful’, those meeting at least 80% of the recommendation were 15, 9, and 3 percentage points higher in males compared to females. For the ‘small handful’ and the ‘30 g serving’, the proportion of males and females was within 1 percentage point.

**Figure 1:**
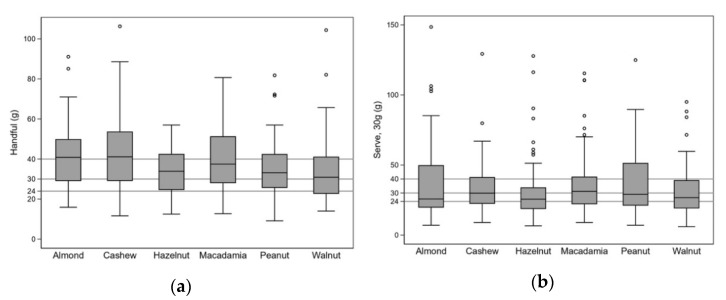
The distribution of portion size chosen, with lines depicting 24 g, 30 g and 40 g, when asked to select: (**a**) a handful; (**b**) an estimated 30 g serving. Hollow circles (⚬) indicate outliers (here defined as more than 1.5 IQR from the nearest quartile).

Overall, a higher proportion of participants chose amounts that exceeded the recommended intake by greater than 10 g (i.e., more than 40 g) when taking a ‘handful’ compared to a ‘30 g serving’ (Figure 1). There was some variation by nut type. A similar proportion chose amounts in excess of 10 g of the recommended intake for almonds and walnuts for both measures, whereas, for cashews, hazelnuts, and macadamias, the proportion was higher for the ‘handful’, and peanuts was higher for the ‘30 g serving’.

Overall, more participants chose at least 80% of the recommended 30 g intake when taking a ‘handful’ compared to a ‘30 g serving’. However, a higher proportion of individuals chose amounts that were at least 10 g higher than the recommended intake when choosing a ‘handful’ compared to a ‘30 g serving’.

### 3.4. The Estimated Percentage of Total Energy Requirement Contribution by Nuts with the Selection of Different Portion Measures

Among all participants when considering all nut types, the median percent contribution of nuts to total energy requirements (TER) when asked to take an estimated ‘30 g serving’ was 12.4% (748 kJ) (10.0% (730 kJ) for males and 13.7% (754 kJ) for females) (Table 4). Conversely, a handful equated to 15.8% of TER (987 kJ) (15.0% (1033 kJ) for males and 16.6% (931 kJ) for females). This increased to 26.2% TER (1630 kJ) (26.2% (1900 kJ) for males and 26.1% (1497 kJ) for females) when asked to take a large handful. A small handful equated to 7.3% TER (449 kJ) (6.4% (468 kJ) for males and 7.8% (427 kJ) for females). A ‘usual serving’ contributed a median of 10.5% to TER (657 kJ) (10.7% (772 kJ) for males and 10.5% (604 kJ) for females).

There was some numerical variation by nut type. For all estimates, excluding the ‘usual serving’, macadamias contributed the highest percentage to TERs, and peanuts the lowest. This was apparent for both genders, except for the ‘30 g serving’ in females, where the contribution to TER was lowest for almonds and hazelnuts.

### 3.5. Predictor of the Different Portion Estimates

From the regression models, there were few statistically significant differences in terms of participant characteristics. BMI and usual consumption were not associated with any of the serving sizes. Using all nut types, males selected larger amounts for their ‘usual serving’ (69.9% higher, 95% CI 17.7–145.1%, *p* = 0.005), older people selected smaller amounts for a ‘handful’ (2.0 g less per 10 years of age, 95% CI 0.2–3.9, *p* = 0.028), taller people selected smaller amounts for their ‘usual serving’ (17.2% less per 10 cm, 95% CI 0.4–31.1%, *p* = 0.045) but more for a ‘large handful’ (5.8 g per 10 cm, 95% CI 0.6–11.0, *p* = 0.030), and people who reported greater hunger selected more for their ‘small handful’ (0.7 g per 10 mm, 95% CI 0.1–1.2, *p* = 0.014).

Exploratory analysis of these results found evidence that the gender effect for ‘usual serving’ varied by nut type (interaction *p* = 0.047), with males selecting more than females for all nut types except for almonds and hazelnuts. The height effects varied by nut type (‘usual serving’ interaction *p* = 0.020, ‘large handful’ interaction *p* < 0.001) with evidence of the association seen for ‘usual serving’ only with hazelnuts and macadamias and for ‘large handful’, only for almonds, cashews, macadamias, and peanuts. There was no evidence for effect modification for the other two results (both interaction *p* ≥ 0.416).

## 4. Discussion

Current nut consumption guidelines in New Zealand and many other countries recommend consuming 30 g of nuts (or 1 ounce, i.e., 28.4 g) per day [5,6,7]. To provide a practical guide, it is often suggested that a 30 g serving is about equal to a handful [8]. To the best of our knowledge, this is the first study to assess the usefulness of handful portions in guiding recommended nut intakes. Overall, the median ‘handful’ serving (36.3 g) resulted in a greater quantity of nuts selected compared to when participants were asked to take an estimated ‘30 g serving’ (28.7 g). When asked to take a ‘handful’ of nuts, the median portion for all nut types was above the recommended 30 g serving, but within 11 g of this guideline. In comparison, when asked to take a ‘30 g serving’, the median intakes for all types were less than the recommended intake, except for macadamias. We also calculated the percentage of participants who chose an amount that was at least 80% of the recommended intake. This was substantially higher when asked to take a handful (83.0%), compared to when asked to take a ‘30 g serving’ (62.7%). Therefore, the portion guide of taking a ‘handful’ of nuts resulted in more individuals taking a quantity which was better aligned with current nut recommendations. However, it should be noted that a small number of individuals chose portions that were particularly large or small. It is clear that some individuals would require more assistance for a handful to be a useful guide.

It should also be noted that when asked to take a ‘handful’, a higher proportion of participants chose servings in excess of 40 g (10 g more than the recommendation), compared to when they were asked to take a ‘30 g serving’. Research suggests that the regular consumption of nuts reduces total and LDL cholesterol in a dose-dependent manner [18]. Therefore, the intake of higher doses of nuts is not a concern for these cardiovascular disease risk factors. However, the high fat and calorie content of nuts mean that larger portions could theoretically result in unwanted weight gain. Yet, most observational studies suggest that regular nut consumers are leaner than non-nut consumers [19]. Intervention studies agree with this observation [20]. A meta-analysis of 33 controlled clinical trials showed that consuming a nut-containing diet did not increase body weight, body mass index, or waist circumference compared with control diets [20]. Other more recent meta-analyses support these findings [21,22].

Three commonly reported mechanisms for the lack of weight gain with regular nut consumption include dietary compensation due to increased satiety, loss of metabolisable energy, and increased energy expenditure. Firstly, nuts are high in fibre and protein, which are two nutrients that promote satiety. A review showed that when participants regularly consume nuts, they make adjustments to their total diet, compensating for around 65–75% of energy provided by the nuts [23]. The crunchy texture of nuts also may enhance satiety due to the mechanical effort required for mastication [24,25]. Secondly, research has shown that the cell walls of nuts may limit the bioaccessability of fat within nuts [26,27]. The incomplete release of fat during digestion has been observed in several studies and it has been estimated that the energy available from nuts is 5–32% lower than predicted from Atwater factors [28,29,30,31,32]. The degree of mastication will also affect the digestibility of nuts, and thus, the amount of metabolisable lipid available [33,34]. A final explanation for the lack of weight gain is the fact that nuts are rich sources of cis-unsaturated fatty acids, in particular, monounsaturated fatty acids, which have high-fat oxidation rates and thus, may increase metabolic rate [35,36,37].

We have previously shown that intakes of up to 60 g of nuts per day over 12 weeks does not adversely affect fat mass in overweight and obese individuals [38]. This suggests that individuals may compensate for at least 60 g. Interestingly, the median amount when asked to take a ‘large handful’ was 61.3 g. Asking people to take a ‘large handful’ resulted in 98.1% of participants consuming at least the recommended nut intake, but also a high proportion choosing far in excess of the recommendation. On average, this may not result in unwanted weight gain if sufficient compensation occurs; however, we should remain mindful that the median energy content of the ‘large handful’ was 1630 kJ, with the contribution to total energy estimated around 26.2%.

Many research groups have designed studies where nuts contribute a specific percentage of total energy [39,40,41,42,43,44,45,46]. This is based on the premise that different sized people, with different activity levels, may benefit from different quantities of nuts. In these studies, the percent energy from nuts has mostly ranged from 10% to 20% [39,40,41,42,43,44,45,46]. Nut recommendations based on TER may make sense from a health perspective, but this is not a useful guide for the general public. These need to be translated into practical servings. In the present study, a handful equated to around 15.8% of TER, 15.0% for males and 16.6% for females. Conversely, a large handful translated to around 26.2% of TER and a small handful to 7.3%. If future research suggests that nut recommendations should be individualised to energy requirements, the measures used in this study may provide useful guides for this approach.

There were some differences between nut types. For example, when participants were asked to take a portion size reflective of their usual intake, median quantities ranged from 16.9 g for hazelnuts to 33.0 g for cashew nuts. This may have reflected preference for the different nut types. Hazelnuts are not commonly consumed in NZ [47]. The most commonly consumed nuts in NZ are almonds, cashews and peanuts [47], which interestingly, were the three nut types with the greatest quantity chosen and better aligned to NZ recommendations, when asked to take a usual serving. The usual macadamia portion was also lower compared to the other nut types. Macadamias are one of the most expensive of the nut types in New Zealand, and this may have influenced portion size choice. A further reason for the difference in the usual quantity could be perceived or expected satiety. Research has shown that consumer perceptions of satiety are affected by psychological factors as well as the physiological properties of a food [48]. Brunstrom et al. have also shown that expected satiety may be more important than liking in guiding portion size [49]. To the best of our knowledge, no studies have compared perceived or expected satiety of different nut types. This would be interesting to examine in future studies.

There were also differences between nut types when asked to take a handful. The amounts of almonds, cashews, and macadamias were higher than hazelnuts, peanuts, and walnuts. We speculate that the density of nuts may influence volume and thus, handful measures. Whereas the density for almonds, cashews, and macadamias are higher compared to walnuts and hazelnuts, this is not the case for peanuts. Morphological differences between nut types may have also influenced portion size selection.

There were some gender differences. Overall, the median ‘handful’ for men was higher than for women; however, this did differ between nut types. When we looked at the predictors of usual portion size, males had a 70% higher portion. This might reflect physical differences in hand size, beyond those explained by height, which was also included in the model, and higher energy requirements, beyond those explained by height and BMI. Gibson et al. also noted gender differences when using hand measures [12]. Male fists had a greater volume of around 100 mL compared to females. Interestingly, the median portion when taking the estimated ‘30 g serving’ was slightly greater among females. Almiron-Roig et al. showed greater error among males when estimating portion size, and they significantly underestimate portions compared to females [50].

We did not find evidence that any servings varied by usual consumption or BMI, or by hunger (aside from ‘small handful’). These results are unexpected and should be interpreted with caution until they can be replicated.

Many food frequency questionnaires use specific serving sizes such as 1 ounce (28.3 g) [10]. In the present study, when we asked participants to choose a 30 g serving, the median for the different nut types ranged from 25.6 g to 31.1 g. This suggests, on average, this is a reasonably useful estimate for population intakes; however, the wide range we observed means that some individual intakes will be grossly under- or over-estimated. However, an FFQ is designed to assess population intakes, and therefore, a 30 g portion is a useful measure for this dietary assessment tool.

There are a number of limitations which should be considered when interpreting the findings. We did not measure the hand size of individuals, and so cannot draw any conclusions about the relationship between portion size and hand size, although height will be correlated with hand size [15,16]. Our sample was not representative of the NZ population, with a higher percentage of females, and predominantly Caucasian and Asian. Additionally, we did not measure the PAL for each individual and instead, used the value for light activity as a conservative estimate of energy requirements.

## 5. Conclusions

A ‘handful’ resulted in a high proportion of individuals taking at least 80% of the recommended intake of nuts. Therefore, this can be used as a practical guide for achieving recommended nut intakes. The median intake when asked to take an estimated ‘30 g serving’ was 28.7 g, which indicates that this is a useful portion size for estimating population nut intakes using FFQs.

## Figures and Tables

**Table 1:** Characteristics of study participants (*n* = 120).

Demographic	
Gender	
Males *n* (%)	45 (37.5)
Females *n* (%)	75 (62.5)
Age (y) ^†^	34.0 (24.0–51.0)
Height (cm)	167.6 (9.8)
Weight (kg) ^‡^	67.1 (1.3)
BMI (kg/m^2^) ^‡^	24.0 (1.2)
Ethnicity *n* (%)	
NZ European	58 (48.3)
Māori	7 (5.8)
Pacific Island	2 (1.7)
Asian	50 (41.7)
Other	3 (2.5)

Values are number (%) unless otherwise stated; ^†^ median (25th and 75th percentiles); ^‡^ geometric mean (geometric sd).

**Table 2:** Median (IQR) quantity of nuts in grams selected for the different portion measures.

Nut Type	Usual Serving			Handful			Large Handful			Small Handful			30 g Serving		
	All	Males	Females	All	Males	Females	All	Males	Females	All	Males	Females	All	Males	Females
Almond	27.3 (31.6) ^a^	32.3 (26.3)	26.0 (31.0)	40.8 (21.0) ^a^	38.8 (18.3)	41.8 (20.6)	66.3 (31.0) ^ab^	78.2 (37.4)	63.5 (22.5)	17.1 (10.5) ^a^	18.7 (10.0)	16.6 (8.4)	25.8 (30.3) ^ab^	24.6 (17.8)	28.1 (39.0)
Cashew	33.0 (36.5) ^a^	41.1 (65.7)	31.8 (34.7)	41.1 (24.7) ^a^	45.0 (21.5)	38.2 (25.3)	70.8 (37.7) ^b^	82.3 (50.6)	66.8 (40.2)	18.9 (13.9) ^b^	18.7 (9.4)	19.1 (16.5)	29.9 (19.1) ^ab^	29.5 (27.2)	30.0 (14.8)
Hazelnut	16.9 (15.4) ^c^	16.4 (16.0)	17.6 (19.5)	33.9 (18.0) ^b^	41.5 (24.5)	32.5 (15.7)	52.9 (25.5) ^c^	66.4 (31.6)	47.9 (15.5)	15.4 (8.4) ^a^	15.7 (8.6)	15.0 (8.0)	25.6 (15.4) ^c^	25.6 (23.1)	25.6 (14.0)
Macadamia	21.6 (32.9) ^ab^	23.4 (22.6)	20.3 (35.3)	37.5 (23.4) ^a^	41.8 (20.2)	34.1 (17.8)	68.2 (24.2) ^a^	74.1 (37.4)	62.3 (24.8)	19.6 (10.2) ^c^	21.1 (10.3)	19.0 (10.0)	31.1 (19.7) ^b^	31.3 (16.6)	31.0 (33.1)
Peanut	29.2 (31.5) ^a^	44.3 (33.1)	24.4 (32.9)	33.1 (16.9) ^b^	35.7 (15.8)	32.5 (19.9)	58.3 (20.2) ^d^	67.8 (23.9)	54.5 (19.9)	14.6 (9.9) ^a^	16.6 (8.5)	13.8 (10,6)	29.1 (30.5) ^b^	27.4 (26.9)	30.2 (34.0)
Walnut	25.9 (31.3) ^b^	32.1 (34.8)	22.2 (27.6)	30.9 (18.6) ^b^	32.1 (17.8)	30.2 (19.5)	56.7 (32.2) ^d^	58.5 (36.9)	54.3 (30.5)	15.2 (9.6) ^a^	15.9 (8.6)	14.7 (10.2)	26.7 (20.2) ^ac^	26.6 (16.3)	26.7 (20.1)
Overall	24.8 (32.7)	30.4 (31.0)	22.4 (32.3)	36.3 (21.2)	39.9 (21.5)	34.2 (19.8)	61.3 (29.2)	71.8 (34.8)	55.5 (26.3)	16.7 (10.0)	17.9 (9.6)	15.7 (10.5)	28.7 (21.2)	28.2 (18.6)	29.0 (21.7)

For the entire sample (both males and females combined), values with different superscript letters are significantly different from each other at the 0.05 level in the linear mixed model looking at nut type, adjusting for the sequence of the measurement task (first, second, or third), gender, age, BMI, height, hunger rating, and how often they consumed that nut type, along with a random effect for participant. For these models, ‘usual’ and ‘30 g serving’ were log-transformed due to skew in the model residuals. For the different portions, participants were asked to take from a large bowl of nuts: (1) what they perceived as a ‘usual serving’ of nuts, (2) a ‘handful’ of nuts, (3) a ‘small handful’, (4) a ‘large handful’ of nuts, and (5) what they believed is equal to a ‘30 g serving’.

**Table 3:** Percentage of participants selecting at least 80% of the recommended intake (30 g) for the different portion measures.

Nut Type	Usual Serving			Handful			Large Handful			Small Handful			30 g Serving		
	All	Males	Females	All	Males	Females	All	Males	Females	All	Males	Females	All	Males	Females
Almond	65.0	77.3	57.9	90.0	90.9	89.5	100	100	100	26.7	36.4	21.1	53.3	50.0	55.3
Cashew	64.4	72.7	59.5	85.0	95.7	78.4	98.3	100	97.3	33.3	26.1	37.8	71.7	73.9	70.3
Hazelnut	29.3	27.8	30.0	78.3	84.2	75.6	98.3	100	97.6	15.0	21.1	12.2	58.3	57.9	58.5
Macadamia	37.3	42.3	33.3	90.0	96.3	84.8	100	100	100	36.7	33.3	39.4	66.7	63.0	69.7
Peanut	62.1	76.2	54.1	81.7	86.4	78.9	96.7	100	94.7	13.3	4.5	18.4	63.3	54.5	68.4
Walnut	53.4	71.4	43.2	72.9	77.3	70.3	94.9	100	91.9	18.6	18.2	18.9	62.7	72.7	56.8
Overall	52.0	61.5	46.4	83.0	88.9	79.5	98.1	100	96.9	24.0	23.7	24.1	62.7	62.2	62.9

For the different portions, participants were asked to take from a large bowl of nuts: (1) what they perceived as a ‘usual serving’ of nuts, (2) a ‘handful’ of nuts, (3) a ‘small handful’, (4) a ‘large handful’ of nuts, and (5) what they believed is equal to a ‘30 g serving’.

**Table 4:** Median (IQR) estimated percentage of total energy requirements provided from nuts with selection of the different portion measures.

Nut Type	Usual Serving			Handful			Large Handful			Small Handful			30 g Serving		
	All	Males	Females	All	Males	Females	All	Males	Females	All	Males	Females	All	Males	Females
Almond	10.9 (11.4)	11.4 (10.7)	10.6 (11.3)	17.1 (8.0)	14.0 (6.9)	17.9 (10.1)	27.0 (11.2)	26.8 (9.2)	27.0 (11.5)	7.4 (3.8)	6.9 (3.6)	7.7 (3.8)	9.9 (11.4)	9.2 (8.5)	12.5 (16.8)
Cashew	13.4 (14.8)	12.9 (24.6)	13.4 (13.1)	15.8 (8.4)	15.0 (6.3)	16.7 (10.9)	27.9 (16.1)	30.0 (13.8)	26.3 (17.7)	6.8 (5.6)	6.0 (3.8)	8.8 (8.5)	13.0 (7.6)	9.4 (8.3)	13.8 (6.5)
Hazelnut	7.5 (7.4)	6.1 (5.4)	9.2 (9.0)	15.8 (7.3)	15.3 (8.5)	15.9 (7.3)	24.3 (9.7)	25.1 (12.0)	23.7 (10.1)	7.2 (3.9)	6.0 (3.4)	7.6 (3.8)	11.8 (7.0)	10.0 (6.7)	12.5 (7.1)
Macadamia	10.7 (17.1)	10.5 (10.1)	11. 1 (19.2)	19.3 (8.4)	20.2 (6.9)	18.9 (9.3)	32.6 (13.7)	32.6 (15.4)	32.6 (13.2)	10.1 (5.4)	9.8 (5.9)	10.9 (6.1)	15.1 (10.6)	12.4 (7.6)	16.8 (15.2)
Peanut	11.8 (13.2)	13.9 (9.0)	10.3 (14.1)	13.1 (9.0)	11.3 (7.9)	14.3 (8.6)	23.5 (7.3)	23.1 (6.4)	23.5 (7.3)	5.9 (3.2)	5.1 (2.6)	6.3 (4.8)	11.5 (11.1)	8.7 (7.3)	13.1 (15.3)
Walnut	11.5 (16.3)	11.7 (15.4)	11.4 (15.7)	14.8 (8.6)	13.4 (6.6)	15.7 (10.2)	27.0 (17.1)	24.2 (14.2)	28.9 (19.4)	7.4 (4.6)	6.3 (3.3)	7.5 (4.7)	12.6 (9.7)	10.2 (7.3)	14.6 (10.3)
Overall	10.5 (13.8)	10.7 (11.7)	10.5 (14.2)	15.8 (8.9)	15.0 (8.1)	16.6 (9.4)	26.2 (13.7)	26.2 (13.4)	26.1 (13.9)	7.3 (4.7)	6.4 (3.7)	7.8 (5.2)	12.4 (9.5)	10.0 (8.3)	13.7 (10.4)

All values are medians (IQR); A PAL for light activity was used to estimate energy requirements. For the different portion measures, participants were asked to take from a large bowl of nuts: (1) what they perceived as a ‘usual serving’ of nuts, (2) a ‘handful’ of nuts, (3) a ‘small handful’, (4) a ‘large handful’ of nuts, and (5) what they believed is equal to a ‘30 g serving’.

## Data Availability

The data presented in this study are available on request from the corresponding author.
